# Astaxanthin Counteracts Vascular Calcification In Vitro Through an Early Up-Regulation of SOD2 Based on a Transcriptomic Approach

**DOI:** 10.3390/ijms21228530

**Published:** 2020-11-12

**Authors:** Chia-Ter Chao, Hsiang-Yuan Yeh, You-Tien Tsai, Tzu-Hang Yuan, Min-Tser Liao, Jenq-Wen Huang, Huei-Wen Chen

**Affiliations:** 1 Nephrology Division, Department of Internal Medicine, National Taiwan University Hospital BeiHu Branch, Taipei 10845, Taiwan; b88401084@gmail.com (C.-T.C.); halukatarosha@gmail.com (Y.-T.T.); 2 Geriatric and Community Medicine Research Center, National Taiwan University Hospital BeiHu Branch, Taipei 10845, Taiwan; 3 Graduate Institute of Toxicology, National Taiwan University College of Medicine, Taipei 100233, Taiwan; shwchen@ntu.edu.tw; 4 School of Big Data Management, Soochow University, Taipei 11102, Taiwan; hyyeh.richard@gmail.com; 5 Genome and Systems Biology Degree Program, Academia Sinica, Taipei 11529, Taiwan; yuan.tzu.h@gmail.com; 6 Department of Pediatrics, Armed Force Taoyuan General Hospital, Taoyuan County 32551, Taiwan; liaoped804h@yahoo.com.tw; 7 Nephrology division, Department of Internal Medicine, National Taiwan University Hospital YunLin Branch, YunLin County 640203, Taiwan

**Keywords:** astaxanthin, aortic calcification, chronic kidney disease, chronic kidney disease-mineral bone disorder, oxidative stress, reactive oxygen species, senescence, vascular calcification, vascular smooth muscle cells

## Abstract

Vascular calcification (VC) is a critical contributor to the rising cardiovascular risk among at-risk populations such as those with diabetes or renal failure. The pathogenesis of VC involves an uprising of oxidative stress, for which antioxidants can be theoretically effective. However, astaxanthin, a potent antioxidant, has not been tested before for the purpose of managing VC. To answer this question, we tested the efficacy of astaxanthin against VC using the high phosphate (HP)-induced vascular smooth muscle cell (VSMC) calcification model. RNAs from treated groups underwent Affymetrix microarray screening, with intra-group consistency and inter-group differential expressions identified. Candidate hub genes were selected, followed by validation in experimental models and functional characterization. We showed that HP induced progressive calcification among treated VSMCs, while astaxanthin dose-responsively and time-dependently ameliorated calcification severities. Transcriptomic profiling revealed that 3491 genes exhibited significant early changes during VC progression, among which 26 potential hub genes were selected based on closeness ranking and biologic plausibility. SOD2 was validated in the VSMC model, shown to drive the deactivation of cellular senescence and enhance antioxidative defenses. Astaxanthin did not alter intracellular reactive oxygen species (ROS) levels without HP, but significantly lowered ROS production in HP-treated VSMCs. SOD2 knockdown prominently abolished the anti-calcification effect of astaxanthin on HP-treated VSMCs, lending support to our findings. In conclusion, we demonstrated for the first time that astaxanthin could be a potential candidate treatment for VC, through inducing the up-regulation of SOD2 early during calcification progression and potentially suppressing vascular senescence.

## 1. Introduction

Vascular calcification (VC) is an important complication that occurs frequently in patients of advanced age, with diabetes mellitus (DM), or chronic kidney disease (CKD) [1]. The ectopic deposition of calcium apatite within the vascular wall is associated with increasing vascular stiffness, impaired aortic compliance, and escalating central blood pressure, leading to compromised coronary perfusion, ventricular compensatory hypertrophy, and a rising risk of cardiovascular mortality [2,3]. VC also serves as an occult cardiovascular risk factor among the general population; a large cohort study involving more than 20,000 young to middle-age adults revealed that screening-identified VC was associated with a ~10-fold increase in the incidence of coronary heart disease-related mortality [4]. Traditional risk factors for atherosclerosis, including smoking, hypertension, and DM, are the main driving forces for developing VC [5]; however, non-traditional risk features, especially the presence of a metabolically noxious environment such as uremic toxins, dysregulated divalent ions, and altered vitamin D/parathyroid hormone statuses, further aggravates an individual’s susceptibility to VC [6]. The complexity of risk features inherent to VC hampers the discovery of efficacious therapeutic options for VC, especially among patients with CKD or DM, who sustain the highest risk.

The pathogenesis of VC has evolved from passive calcium deposition to the involvement of active osteoid secretion by trans-differentiated vascular smooth muscle cells (VSMCs). Triggering of such phenotypic changes is multi-faceted, including inflammatory stimuli, excessive calcium and/or phosphate exposure, prolonged exposure to pro-calcific and pro-fibrotic mediators, and so on, assisted simultaneously by the decline in anti-calcific defending molecules [7]. Specifically, oxidative stress induces and accelerates the progression of VC through modulating RUNX2 expressions, the core fate-determining effector of osteoblastic switch [8]. Sources of reactive oxygen species (ROS) ranging from peroxides, advanced oxidized protein products (AOPPs), to advanced glycation endproducts (AGEs) have all been shown to precipitate bio-mineralization among different types of cells subject to adverse stimuli [9]. From this perspective, anti-oxidants are purported as promising therapeutic options for managing VC of diverse origins [10].

Astaxanthin, a type of carotenoid derivative predominantly found in the red pigments of crustacean shells and salmons, is a natural color additive frequently used for salmon flesh quality enhancement. Discovered in 1938 from lobsters, astaxanthin has long been reported to manifest an anti-oxidative capacity thanks to its chemical structure, consisting of an extended conjugated carbon chain with polar regions at both ends [11]. This unique structure endows astaxanthin with a free radical scavenging ability both in vitro and in vivo. Prior reports disclosed that astaxanthin possessed 100- to 500-fold higher antioxidant activity compared with vitamin E, β-carotene, and other carotenoids such as zeaxanthin and lutein [12]. However, available studies have not examined whether astaxanthin may exert beneficial effects against VC, and if present, what would be the underlying molecular machineries. In this study, we tested the efficacy of astaxanthin for treating VC and harnessed a transcriptomic approach to systematically investigate the associated mechanisms.

## 2. Results

### 2.1. Evaluating the Effect of Astaxanthin in the In Vitro VC Model

**Figure 1:**
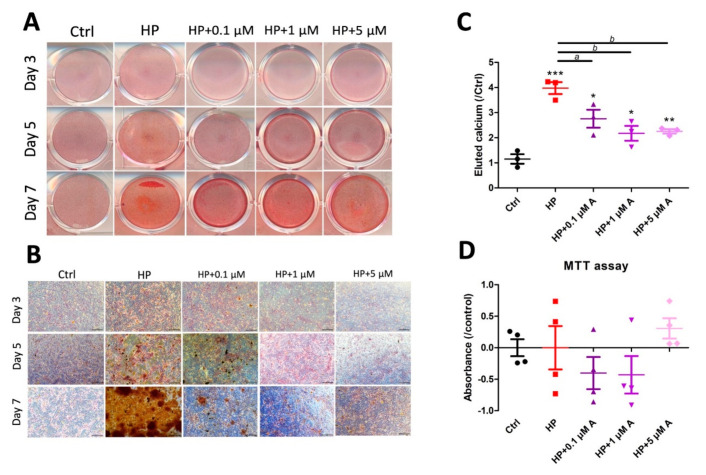
Selecting the optimal experimental condition for astaxanthin in VC models. (**A**) Macroscopic and (**B**) microscopic examination of vascular smooth muscle cells subjected to DMEM or HP media with different concentrations of astaxanthin over 3, 5, and 7 days. Scale bar, 200 μm. (**C**) Quantitation of calcification in vascular smooth muscle cells with and without escalating concentrations of astaxanthin. (**D**) Viability assay results of cells with and without escalating concentrations of astaxanthin. * *p* < 0.05, ** *p* < 0.01, and *** *p* < 0.001 compared with Ctrl; ^a^ *p* < 0.05 and ^b^ *p* < 0.01 compared with HP. A, astaxanthin; Ctrl, control; DMEM, Dulbecco’s Modified Eagle’s Medium; HP, high phosphate; VC, vascular calcification; MTT, 3-(4,5-dimethylthiazol-2-yl)-2,5-diphenyltetrazolium bromide.

We first examined the efficacy of high phosphate (HP) on the cultured VSMCs over time. HP induced progressive VSMC calcification over 7 days of culture, presenting as an increasing extent of positive Alizarin red staining areas both macroscopically (Figure 1A) and microscopically (Figure 1B). We showed that higher concentrations of astaxanthin dose-dependently attenuated the severity of calcification over time, with less Alizarin red positive nodules in cells exposed to higher doses (Figure 1A,B). We discovered that 0.1, 1, and 5 µM of astaxanthin abolished 33%, 70%, and 67%, respectively, of calcification severity compared with the HP only group after 7 days of treatment (Figure 1C). No significant differences in calcification severities were noted between the 1 and 5 µM groups. Besides, increasing concentrations of astaxanthin did not negatively influence cell viability over 7 days of treatment (Figure 1D).

### 2.2. Microarray Experiments for Investigating Mechanisms Behind the Astaxanthin Effect

**Figure 2:**
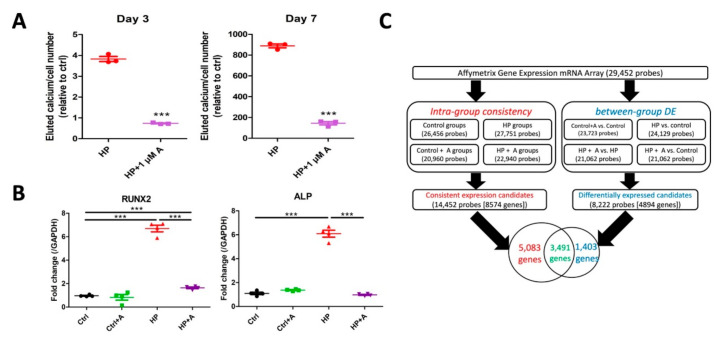
Microarray experiments and the bioinformatic workflow. (**A**) Quantitation of calcification in cells subjected to HP and HP + astaxanthin at day 3 (left) and 7 (right). (**B**) Osteoblastic differentiation genes’ (left, RUNX2; right, alkaline phosphatase (ALP)) expression levels in cells undergoing different treatments. (**C**) The algorithm of sequentially analyzing transcriptomic data generated from Affymetrix microarray experiments. *** *p* < 0.001 compared with the reference group; A, astaxanthin; Ctrl, control; DE, differential expression; HP, high phosphate.

We next selected the optimal timing and concentrations of astaxanthin for treating VSMCs, in order to uncover mediators of astaxanthin effect on VC. From Figure 1C, it is evident that the maximal therapeutic efficacy of astaxanthin against calcification could be achieved when 1 µM was used. In addition, according to the literature, pathogenesis of VC may vary between the early and the late phase of progressive calcification [13], and molecular alterations occurring during the first 3 days of in vitro calcification are more reflective of the direct effects of noxious injuries [13,14]. Consequently, we opted to evaluate samples from HP-treated VSMCs exposed to astaxanthin at day 3 for subsequent microarray experiments. We showed that 3 and 7 days of HP exposure induced significantly greater VSMC calcification over time than control cells, while 1 µM of astaxanthin induced nearly 80% and 85% reduction of calcification amount, respectively (Figure 2A), supporting that 3 days of HP already produced sufficient calcification extent and permitted mechanistic workup at an early stage of VC. VSMCs exposed to 3 days of HP led to significantly higher expressions of RUNX2 and alkaline phosphatase (ALP), while astaxanthin treatment completely abolished such influences (Figure 2B). Astaxanthin did not alter RUNX2 and ALP expressions without HP exposure among VSMCs (Figure 2B).

**Figure 3:**
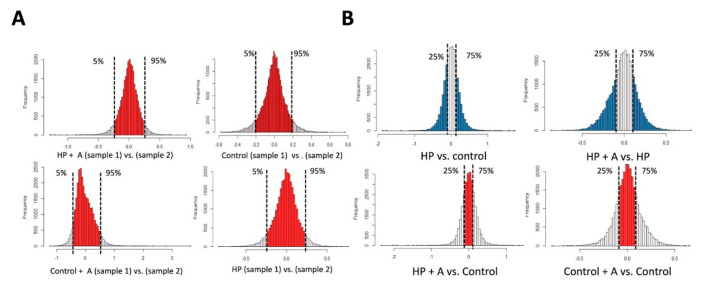
Distribution plots of all examined probes upon intra-group and inter-group comparisons. (**A**) The illustrations of intra-group comparisons between biological duplicates of HP + A (left upper), control (right upper), control +A (left lower), and HP (right lower). (**B**) Inter-group comparisons between HP and control (left upper), between HP + A and HP (right upper), between HP +A and control (left lower), and between control + A and control (right lower). A, astaxanthin; HP, high phosphate.

RNAs collected from biological duplicates of control and HP-treated VSMCs with and without astaxanthin exposure were first checked for purity and stability. After ensuring an RNA integrity number (RIN) of at least 7, samples were subjected to Affymetrix mRNA array, containing 29,452 probes. The subsequent bioinformatic workflow is demonstrated in Figure 2C. In brief, we first checked the intra-group consistency (Figure 3A) and between-group differential expressions (Figure 3B) based on pre-specified thresholds. The distributions of probe readouts were normalized beforehand. For differential expressions, only those exhibiting up-regulation upon HP treatment followed by down-regulation upon astaxanthin addition (astaxanthin-responsive VC enhancers), or those exhibiting down-regulation upon HP treatment followed by up-regulation upon astaxanthin addition (astaxanthin-responsive VC suppressors), were selected for functional enrichment analysis and validation (Figure 3B, upper row). Another criterion of expression level restoration by astaxanthin, presenting as an insignificant difference between levels in control groups and those in HP + astaxanthin group, was imposed to narrow down the candidate list (Figure 3B, left lower panel). In total, 8574 and 4894 genes exhibited consistent expression levels and had differential expressions upon astaxanthin exposure during the HP condition, respectively, among which 3491 (25.9%) shared both features (Figure 2C).

**Figure 4:**
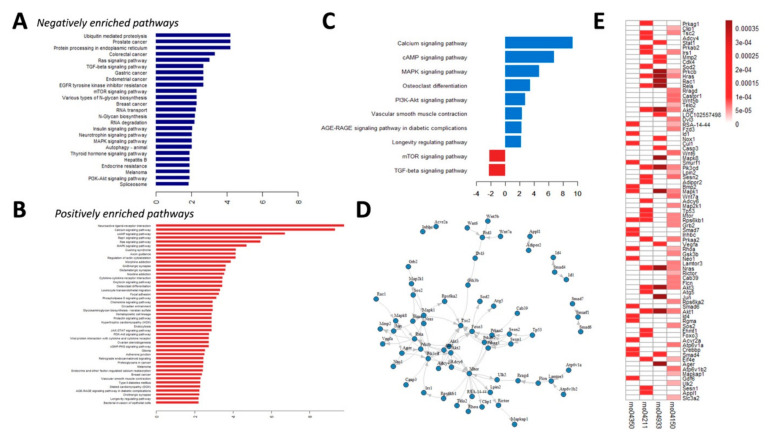
Pathways enrichment analysis. KEGG pathways with negative (**A**) and positive (**B**) changes upon astaxanthin treatment were listed, followed by literature- and biological plausibility-based trimming, with core processes (**C**) displayed. (**D**) A node plot illustrating the regulatory relationships between candidate genes in core processes listed in panel C. (**E**) A heatmap showing the closeness scores of each gene from the identified core processes described in text. KEGG, Kyoto Encyclopedia of Genes and Genomes; mTOR, mammalian target of rapamycin; AGE, advanced glycation endproduct; RAGE, receptor of AGE; TGF, transforming growth factor.

**Figure 5:**
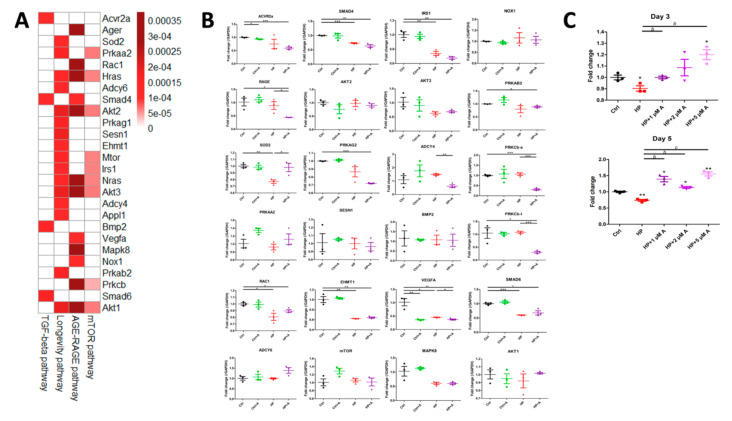
Validation of hub gene expressions in vitro. (**A**) Another heat map showing the closeness scores of the 26 early hub genes. (**B**) Expression levels of early hub genes in VSMCs subjected to HP and control media with and without astaxanthin treatment. (**C**) SOD2 expression levels among VSMCs subjected to control or HP media with escalating concentrations of astaxanthin at day 3 (*upper*) and 5 (lower). * *p* < 0.05, ** *p* < 0.01, and *** *p* < 0.001 compared with Ctrl or the reference group; ^a^ *p* < 0.05, ^b^ *p* < 0.01, and ^c^ *p* < 0.001 compared with the HP group. A, astaxanthin; AGE, advanced glycation endproduct; C, control; Ctrl, control; HP, high phosphate; mTOR, mammalian target of rapamycin; RAGE, receptor of advanced glycation endproduct; TGF, transforming growth factor; VSMC, vascular smooth muscle cell.

The 3491 candidates subsequently underwent functional enrichment, with the results shown in Figure 4A (negatively deregulated) and 4B (positively deregulated). Important pathways including protein processing and metabolism (endoplasmic reticulum processing, proteolysis, mammalian target of rapamycin (mTOR), and transforming growth factor-β (TGF-β) signaling) were suppressed during astaxanthin treatment, while they were induced by HP (Figure 4A); pathways such as calcium, cAMP, and MAPK signaling; cytokine receptor interaction; chemokine signaling; and cytoskeletal regulation were induced during astaxanthin treatment, while they were attenuated by HP (Figure 4B). We further focused on pathways that contributed potentially to VC pathogenesis through biologically meaningful connections based on the existing literature [10,15,16,17], with 79 genes from 8 and 2 positively and negatively enriched pathways, respectively, retrieved (Figure 4C). A node plot illustrating the regulatory networks between pairable candidates among all the enlisted genes is shown in Figure 4D. To explore the strengths of regulatory relationships and identify hub genes, we measured the strengths of each candidate gene within four Kyoto Encyclopedia of Genes and Genomes (KEGG) pathways (mTOR and TGF-β signaling, receptor of AGE (RAGE) signaling, and longevity-regulating pathways) with both *p*-value and q-value < 0.05, using a closeness plot (Figure 4E). Twenty-six potential hub genes were thus selected from the candidate list based on closeness ranking and biologic plausibility, with closeness scores provided (Figure 5A). These hub genes represented pivotal effector molecules in action early during VC pathogenesis that were influenced by HP but were restorable by astaxanthin treatment.

### 2.3. Validation of Potential Early Hub Gene In Vitro

We validated the expression levels of the selected hub genes (except Ras) in VSMCs subjected to HP and astaxanthin treatment (Figure 5B). Expression levels of several early hub genes were suppressed (EHMT1, IRS1, PRKAG2, RAC1, SMAD4, and SMAD6) by the HP condition, but were minimally responsive to astaxanthin treatment. ACVR2a was suppressed by astaxanthin treatment, but not influenced by HP, while ADCY6, AKT1, AKT2, AKT3, BMP2, MAPK8, mTOR, NOX1, PRKAA2, and SESN1 were not influenced by HP nor astaxanthin early during VC. Among the 26 predicted hub genes, only SOD2 exhibited corresponding changes reflective of the astaxanthin effect (Figure 5B); SOD2 expressions were down-regulated during HP exposure, but were completely restored by astaxanthin treatment. We further examined SOD2 expressions in VSMCs subjected to escalating concentrations of astaxanthin at consecutive time points after HP exposure. It is clear that 1 µM of astaxanthin completely abolished HP-induced down-regulation of SOD2, while higher doses (2 and 5 µM) of astaxanthin induced higher SOD2 expressions compared with the 1 µM group at day 3 (Figure 5C, upper). This trend of astaxanthin-induced SOD2 up-regulation became more prominent at day 5 and during higher concentrations of astaxanthin exposure (Figure 5C, lower). Protein levels of SOD2 in VSMCs with and without HP and astaxanthin exposure exhibited the same changes as mRNA levels (control vs. control + astaxanthin vs. HP vs. HP + astaxanthin, 1.0 vs. 1.1 vs. 0.77 vs. 0.88).

### 2.4. Functional Characterization of SOD2 During VC

**Figure 6:**
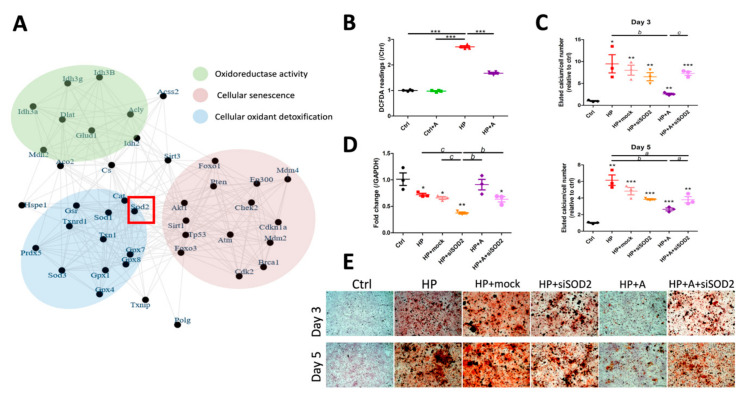
Functional characterization of SOD2. (**A**) A node plot illustrating the interactions between SOD2 and potential effector pathways, divided according to the different spectrum of biologic processes. Red square denoted the validated core hub genes, SOD2. (**B**) VSMC ROS amount depending upon control or HP media with and without astaxanthin treatment. (**C**) Quantitation of calcification in SOD2 wild type or silenced cells with and without HP or astaxanthin exposure. (**D**) SOD2 mRNA expression levels and (**E**) microscopic examination images in each group. Scale bar, 200 μm. * *p* < 0.05, ** *p* < 0.01, and *** *p* < 0.001 compared with Ctrl or the reference group; ^a^ *p* < 0.05, ^b^ *p* < 0.01, and ^c^ *p* < 0.001 for comparison. A, astaxanthin; Ctrl, control; DCFDA, 2′,7′-dichlorofluorescin diacetate; HP, high phosphate; ROS, reactive oxygen species; siSOD2, SOD2 silenced group; VSMC, vascular smooth muscle cell.

To understand the influences related to SOD2 expression changes during VC, we adopted three approaches. First, we dig through the protein–protein interaction network to uncover plausible biological processes associated with VC amelioration. Among the interaction network of SOD2, we discovered that genes influenced by SOD2 modulation were enriched in the following three biological functions: oxidoreductase activity, cellular senescence, and cellular oxidant detoxification (Figure 6A). Second, because SOD2 is a vital antioxidant gene capable of eliminating mitochondrial ROS [18], we examined cellular ROS extent among HP-, astaxanthin-, and co-treated VSMCs. Astaxanthin did not alter intracellular ROS levels without HP exposure, but significantly lowered ROS production in HP-treated VSMCs (Figure 6B). Finally, we examined calcification propensity among astaxanthin-treated VSMCs with SOD2 knockdown, in order to validate the role of SOD2 in mediating the anti-calcification effect of astaxanthin. SOD2 knockdown essentially abolished the anti-calcification effect of astaxanthin on HP-treated VSMCs, while the SOD2 knockdown did not alter the pro-calcification effect introduced by HP without astaxanthin (Figure 6C), and SOD2 was successfully silenced on day 3 (relative expressions of the control vs. HP vs. HP + mock vs. HP + siSOD2 vs. HP + astaxanthin vs. HP + astaxanthin + siSOD2 groups, 1.01 ± 0.21 vs. 0.72 ± 0.04 vs. 0.66 ± 0.05 vs. 0.37 ± 0.04 vs. 0.91 ± 0.17 vs. 0.63 ± 0.09, respectively) (Figure 6D). The calcium quantitation results were also supported by Alizarin red positively stained areas observed microscopically (Figure 6E).

## 3. Discussion

**Figure 7:**
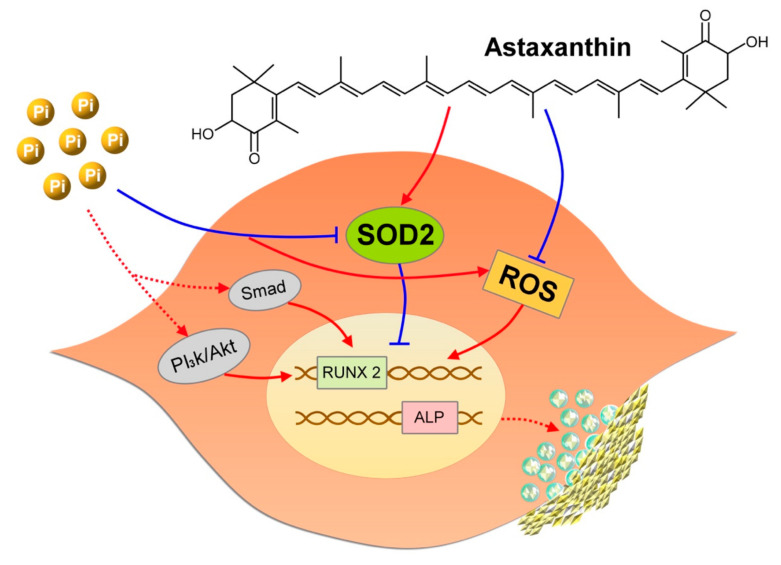
A graphical illustration of findings in this study. ALP, alkaline phosphatase; Pi, inorganic phosphate; ROS, reactive oxygen species; SOD2, superoxide dismutase 2.

In this study, we evaluated the effect of astaxanthin on VC and discovered, for the first time, that astaxanthin decreased the severity of VC and delayed its progression. Through transcriptomic analyses and bioinformatic dissections, astaxanthin exerts its beneficial effect mainly through an early restoration of SOD2 expressions among calcified VSMCs, leading to less ROS production and a lower probability of calcification worsening (Figure 7). Pathways including oxidoreductase, oxidant detoxification, and senescence are potential downstream effectors of SOD2, accounting for the beneficial vascular effects conferred by astaxanthin. Judging from our findings, the clinical applicability of astaxanthin in managing VC may be promising in the future.

The range of astaxanthin concentration exhibiting biological effects can be wide, but the optimal condition varies between diseases and even cell types. A prior review summarized the experimental anti-cancer, hypolipidemic, anti-diabetic, and organ protective effects of astaxanthin, and found that 0.1~100 µM astaxanthin treatment for 24 h to 7 days can be potentially efficacious [12]. With regard to anti-oxidative effect, Wolf et al. reported that 0.8 µM astaxanthin attenuated mitochondrial ROS extent in Hela cells after 1 day of treatment [19]. On the other hand, Campoio and colleagues revealed that 2 µM astaxanthin partially lowered oxidative stress severity, including lower free radical production, interleukin-1β levels, and suppressed myeloperoxidase expressions in human lymphocytes [20]. Higher concentrations of astaxanthin, up to 40 µM, provide even more anti-oxidant ability, but cell viability may be potentially compromised [21]. Our findings that 1 µM astaxanthin exhibited optimal anti-calcific effects through lowering ROS production thus fall within the effective dose range reported by others (Figure 1). We further showed that the 1 µM group did not influence cell viability, which supports the utility of a low astaxanthin dose as a plausible VC treatment option.

The ability of up-regulating anti-oxidant enzymes by astaxanthin has been demonstrated previously, but few addressed the influences of astaxanthin on SOD2 expressions. In a model of renal ischemic-reperfusion injury, astaxanthin treatment restored renal SOD enzyme activities, while it lowered lipid peroxidation severities, but SOD2 levels were not examined [22]. Another study similarly showed that astaxanthin increased SOD enzyme activities in irradiated bone marrow cells without altering the baseline SOD levels [23]. Only one report showed that astaxanthin up-regulated SOD2 expressions in gastric epithelial cells when injured by *Helicobacter pylori* [24]; however, they did not demonstrate the phenotypic changes or clinical relevance of such an effect. None of the existing studies addresses the astaxanthin effect in vascular cells. In this study, we demonstrated that astaxanthin could ameliorate VSMC calcification through up-regulating SOD2 levels and suppressing ROS production early during the course, which is novel and of potential importance. Although our bioinformatic analyses did not pinpoint other antioxidant genes or antioxidant response elements as mediators of the astaxanthin effect, we also checked the mRNA levels of NRF2 and NQO1 on day 3 of our experiments; neither of these two genes exhibited significant changes in expressions (data not shown). This lends support to our microarray findings that astaxanthin might induce an early change in SOD2 even before the onset of feedback loops in other antioxidation responsive genes.

During functional characterization, astaxanthin was found to deactivate cellular senescence pathways through up-regulating SOD2 expressions (Figure 6A). Indeed, oxidative stress has been shown to be an integral predecessor of cellular senescence [25], and vascular aging now emerges as a critical contributor to VC susceptibility [14,16]. Astaxanthin, through its ability of neutralizing ROS and optimizing membrane fluidity, is now recognized to be an anti-aging candidate agent [26] that exerts beneficial effects on the skin, brain, immune system, kidney, and liver, potentially extending the life span of treated animals. Based on our findings, it is highly likely that astaxanthin further promotes vascular health through attenuating vascular aging and dysfunction, with the manifestation of less VC.

The clinical application of astaxanthin can be diverse, judging from the organ protective effects it shows and its established safety profile. Astaxanthin has existed as a food additive for more than two decades, and has been judged by European Medical Agency (EMA) as not harboring mutagenicity, reproductive/developmental toxicity, and clastogenecity [27]. The report from EMA also identified that healthy human volunteers consuming 5 to 12 mg of astaxanthin daily for 4 to 8 weeks did not exhibit intolerance or toxicity. In this sense, we believe that astaxanthin may be well suited as a therapeutic option for VC, with a good safety profile and minimal toxicity concerns.

Our study has its strengths and limitations. Astaxanthin has not been investigated before for treating VC, and we have, as a pilot attempt, demonstrated its utility for improving VC. Our findings thus have extended the therapeutic spectrum of astaxanthin, as the efficacy of available therapeutic approaches for VC is mostly disappointing [10]. The dose- and time-responsive relationships between astaxanthin and the calcification intensity support its biologic plausibility, and the transcriptomic approach we adopted likely captured the whole pathogenic picture early during the process of VC. However, issues that need to be born in mind before interpreting our results include the stage of molecular changes we focused on (early VC) in our experiments and the homogeneity of VC model we tested. Experiments using human VSMCs and in vivo animal models with and without astaxanthin treatment may also be needed to further support our in vitro findings. Nonetheless, astaxanthin has been shown in human cells to restore SOD2 expression levels following the suppression of its expression by noxious stimuli [24]. In addition, molecular alterations induced by astaxanthin have been shown to be consistent across cell models of different species, including mice, rats, and humans [28,29,30]. Our findings are compatible with existing knowledge and are expected to broaden the clinical indications of astaxanthin in the future.

## 4. Materials and Methods

### 4.1. Cell Lines, Reagents, and Experimental Utilities

Rat aortic smooth muscle cells (A7r5) were obtained from American Type Culture Collection (ATCC). Astaxanthin, Alizarin red, and 3-(4,5-dimethylthiazol-2-yl)-2,5-diphenyltetrazolium bromide (MTT) were all purchased from Sigma-Aldrich (St. Louis, MO, USA) and were of analytical grade. The calcium quantitation kit was retrieved from Abcam (ab102505; Cambridge, UK). ROS amounts were measured using the kit from Abcam (ab113851). For knockdown of target genes, we harnessed siRNAs from Dharmacon (ON-TARGET plus SMARTpool, ThermoFisher Scientific, Waltham, MA, USA). Sequences of primers used for quantitative real-time polymerase chain reaction (RT-PCR) of target genes are shown in Table 1. Antibodies used for Western blotting were as follows: primary antibodies against SOD2 and β-actin were from Genetex (GTX116093; Irvine, CA, USA) and Novus (NB600-501; Littleton, CO, USA), respectively, while secondary goat anti-rabbit antibodies were from R&D Systems (HAF008; 1:5000 dilution; Minneapolis, USA). All experiments were carried out with biologic triplicates at least.

### 4.2. VC Model Construction and Calcification Phenotype Assay

The details of in vitro VC model establishment have been illustrated in our prior work [31,32,33]. In brief, VSMCs were subjected to osteogenic media containing 2.5 mM phosphate (HP) for various lengths, followed by periodic phenotypic evaluations. We evaluated VSMC calcification using both qualitative and quantitative approaches at different time points of experimental treatments of escalating dosages. This was conducted in order to determine the optimal dose and time point for determining the condition based on which we subsequently collected RNA samples for profiling. Qualitative examination was done by the Alizarin red staining, which characterized extracellular calcium deposition through calcium chelation followed by macroscopic and microscopic observations. Quantitative examination was done by acid elution of calcified VSMCs, with eluate determined spectrophotometrically for calcium amount and normalized to protein quantity or cell counts. To ascertain whether osteoblastic trans-differentiation occurred, we further used RT-PCR to measure the expressions of ALP and RUNX2.

### 4.3. Cell Viability Assay

To exclude any cytotoxic effect of astaxanthin, we checked VSMC viability after different concentrations of astaxanthin exposure using the MTT assay. In brief, following the removal of media from cultured VSMCs, a fixed amount of MTT solution with serum-free media was added to each well, followed by incubation in room temperature. MTT solvent was later added to cultured VSMCs with gentle shaking, and each well received a spectrophotometric reading at OD 590 nm.

### 4.4. Microarray Experiments and Bioinformatic Analyses for Mechanistic Investigations

After selecting the dose and optimal period of astaxanthin exposure, we collected RNAs from controls, astaxanthin-treated, HP-treated, and HP/astaxanthin-cotreated VSMCs. Expression data were obtained after rendering RNAs from the four groups to transcriptomic profiling based on the Affymetrix GeneChip platform, with group-specific cell intensity files (CELs) generated. In total, eight mRNA expression profiles (biological duplicates for all four groups) were obtained. We applied RMA normalization using the *affy* package of the R software and applied the quantile normalization method to handle CEL files for variance stabilization. We harnessed an intra-group comparison between two replicated samples to examine the consistent expressions among genes to ensure the intra-array consistency. We also conducted an inter-group comparison between the biologic duplicates from different conditions to uncover candidate genes with significant between-array differences. The intra-group consistency and between-group differential expressions of each gene probe were evaluated based on the quantitative signal values as a measure of the gene selection procedure. Low intra-group variability and high between-group differences have been regarded as important properties of the reliable biomarkers [33]. To ensure the presence of both criteria, we adopted the criteria of including genes with fold changes between the 95th percentile and 5th percentile and those with fold changes above the 75th percentile or below the estimated 25th percentile, respectively. The differential expressions of mRNAs were visually displayed in distribution plots, with the x- and *y*-axis denoting fold changes and the number of genes studied, respectively. The identification of consistently differentially expressed genes was followed by functional enrichment analysis and targeted trimming based on the biological relevance to the process of calcification, as candidates uncovered based purely on the ranking of fold differences of expressional levels frequently yielded unrelated genes.

### 4.5. Functional Pathway Enrichment

We further classified differentially expressed genes according to the functional enrichment results. We mapped differentially regulated genes to Entrez Gene database and analyzed the functional enrichment of the biomarkers in specific pathways using clusterProfiler R package with KEGG pathway term enrichment (enrichKEGG) function. We used the hypergeometric testing with more than ten terms enriched in the pathway category and the Benjamin–Hochberg correction method for multiple testing by controlling the false discovery rate (FDR). We calculated minus logarithmically transformed *p*-values for KEGG pathway terms and summarized the results in scores and var plots.

### 4.6. Network Closeness and Measurement

As the differentially expressed genes formed regulatory networks under the control of various factors, we further identified critical hub genes using the interactions of regulatory relationships. To clarify the crosstalk between gene expression and regulatory changes, we applied the network-based method to understand the importance of the genes within the identified pathway. We illustrated important pathways in graphs in which nodes denoted genes and edges denoted interactions. The closeness centrality of a given node measured the centrality of a node based on how close it was to others in the network. The smaller the length of the shortest path between a given node and others, the greater its closeness. Significant hub genes were selected from the network, followed by subsequent validation by RT-PCR and Western blotting in the experimental VC models.

### 4.7. Extended Protein Interaction Network

We further extended the interaction networks for candidate hub genes selected and validated above, based on the protein–protein interactions (PPIs) derived from Search Tool for the Retrieval of Interacting Genes/Proteins v.11 (STRING v.11). We constructed the networks from the validated hub genes with their differentially expressed first and second order neighbors under different regulatory mechanisms. 

### 4.8. Functional Evaluation of Hub Genes

We manipulated the validated hub genes in our experimental models of VC, using VSMCs with knockdown of the hub genes, and observed whether the changes of calcification severity were reversed upon astaxanthin exposure over different durations. Changes in cellular ROS amount were also assessed in VSMCs with and without astaxanthin exposure.

## 5. Conclusions

In conclusion, we used in vitro VC models with and without astaxanthin exposure followed by a transcriptomic approach to uncover potential effector molecules. SOD2 was identified as an early responder to astaxanthin treatment in VSMCs, and the severity of oxidative stress was substantially attenuated. Other pathways targeted by astaxanthin through SOD2 regulation included oxidoreductase activity, cellular senescence, and cellular oxidant detoxification. Astaxanthin may serve as a new and early awaited option within the therapeutic armamentarium against VC.

## Figures and Tables

**Table 1:** Primer sequences used in this study.

Gene	Sequences
ACVR2a	F: AAGTCACACAGCCCACATCA R: GTCCAGGGTCCTGAGTAGGAA
ADCY4	F: GAGCCTAGCTTTGCTACGAG R: GATCGCGCAGAGCACAATG
ADCY6	F: GGCGCAGCAGCATGTCA R: GTGAGGCTGCTCTGGTTCAT
AKT1	F: TACCTGAAGCTACTGGGCAAGGG R: CGGTCGTGGGTCTGGAATGAG
AKT2	F: CTCTGTAGCAGAATGCCAGC R: ATGGAAGGTCCTCTCGATGA
AKT3	F: CCCCCGAACACTCTCTTCAG R: AGTCTTCCTGAAATTGAACCAGAT
ALP	F: TTGGTCTGGCTCCCATGGTG R: GCAAAGACCGCCACATCTTCC
BMP2	F: CACGAGAATGGACGTGCCC R: GCTTCAGGCCAAACATGCTG
EHMT1	F: GGTCAAGTATGAGCTGATGCG R: GTCACTGTGGAAGTCGTGTCTG
GAPDH	F: AACGGCACAGTCAAGGCTGA R: ACGCCAGTAGACTCCACGACAT
IRS1	F: TTCGTGCAGACACTCAAGGC R: TTTAGGTCTTTCATTCTGCCTGT
MAPK8	F: AGCCGGCCATTTCAGAATCA R: CTTGTAGCCCATGCCGAGAA
NOX1	F: CGAAGTGGCTGTACTGGTTG R: AAAGGCACCCGTCTCTCTAC
mTOR	F: CCTCGGCACATCACTCCCTT R: GCTCCTACATTTCAGCACCCACT
PRKAA2	F: GATCGGACACTACGTGCTGG R: CTTCCACCTCTTCAACCCTCC
PRKAB2	F: TGATTCTGTGAAGCCCACCC R: TGACTGGTAACCACAGGCTCT
PRKAG2	F: CAGCACCGGTCACACTACTT R: CAGACGGTGAACCTCTGCTT
PRKCb-s	F: CATCCACCAGTCCTAACACCT R: TGTTGAGCTTGAATGACAGAAAT
PRKCb-l	F: GAACTGACTCCCACTGACAAACT R: CACCATGAATCCTGGAAGACT
RAC1	F: CCTACCCGCAAACAGACGTG R: ACACTGTCTTGAGTCCTCGC
RAGE	F: GTCTGGGCTGGGAACGCTAG R: TGAAGGAGACAGGGTTTGCC
RUNX2	F: TCTTCCCAAAGCCACAGCG R: TGCCATTCGAGGTGGTCG
SMAD4	F: TTCACTGCTCAGCCAGCTAC R: TCATTGTGAACCGGCCAGTAA
SMAD6	F: CCGGGTGAATTCTCAGATGCC R: TGCTGACAGCCTGGTCATAC
SESN1	F: AGGAAGTGCCTTGGTGAGTGA R: TGGAACATGCATCCCATGCC
SOD2	F: ACGCGACCTACGTGAACAAT R: TAACATCTCCCTTGGCCAGC
VEGFA	F: GCAGAAAGCCCATGAAGTGGT R: TGCTGCAGGAAGCTCATCTC

F: forward; R: reverse.

## Abbreviations

AGE	advanced glycation endproduct
ALP	alkaline phosphatase
ATCC	American Type Culture Collection
AOPP	advanced oxidized protein product
CEL	cell intensity file
CKD	chronic kidney disease
DM	diabetes mellitus
EMA	European Medical Agency
HP	high phosphate
KEGG	Kyoto Encyclopedia of Genes and Genomes
mTOR	mammalian target of rapamycin
MTT	3-(4,5-dimethylthiazol-2-yl)-2,5-diphenyltetrazolium bromide
PPI	protein-protein interaction
RAGE	receptor of advanced glycation endproduct
RIN RNA	integrity number
ROS	reactive oxygen species
RT-PCR	real-time polymerase chain reaction
STRING	Search Tool for the Retrieval of Interacting Genes/Proteins
TGF-β	transforming growth factor-β
VC	vascular calcification
VSMC	vascular smooth muscle cell
